# Embryonal rhabdomyosarcoma of the perineum in an adult: a case report

**DOI:** 10.1186/s13256-016-1166-2

**Published:** 2016-12-20

**Authors:** Sidy Ka, Freddy Gnangnon, Mamadou Moustapha Dieng, Doudou Diouf, Jaafar Thiam, Pape Macoumba Gaye, Ahmadou Dem

**Affiliations:** Joliot Curie Institute, Dakar, Senegal

**Keywords:** Embryonal, Rhabdomyosarcoma, Adult, Immunohistochemistry

## Abstract

**Background:**

We report the case of an adult patient with embryonal rhabdomyosarcoma of the perineum admitted to our practice at Joliot Curie Institute in Dakar. It is a rare tumor at this age and has a bad prognosis at this localization.

**Case presentation:**

We describe the case of a 22-year-old African man admitted for a perineal mass that had evolved over 6 months. He complained of tenesmus, obstinate constipation, and dysuria. A clinical examination revealed perineal swelling spread over his anus, scrotum, penis, testicles, and inguinal lymph nodes. A perineal ultrasound and computed tomography scan showed a large mass driving his testicles forward with regional lymph node metastases. An ultrasound-guided biopsy showed embryonal rhabdomyosarcoma on histology and immunohistochemistry, with strong positivity of neural cell adhesion molecule and myogenin while results for cytokeratin AE1/AE3, cluster of differentiation 45, synaptophysin, and chromogranin were negative. Our patient was classified T2N1M1. Outcome was quickly marked by occlusive syndrome and colostomy. Our patient did not opt for chemotherapy and died after 6 months of follow-up.

**Conclusions:**

The embryonic RMS of the adult is a rare disease. Despite the sensitivity to chemotherapy and surgery. Localization to perineum remains poor prognosis.

## Background

Embryonal rhabdomyosarcoma (ERMS) is a rare type of soft tissue tumor [1]. It predominates in children less than 7 years old while a second peak is observed in adolescence; the two subtypes that affect both child and adult are embryonal and alveolar rhabdomyosarcomas. A third subtype, the pleomorphic RMS occurs almost exclusively in adults [2]. The most common sites are cervical-cephalic and genitourinary [3]. Perineal and anal localizations are rarer and have worse prognosis [4].

We report the case of a 22-year-old patient with ERMS of the perineum extended at the scrotum and penis with inguinal lymph node involvement.

## Case presentation

**Fig. 1:**
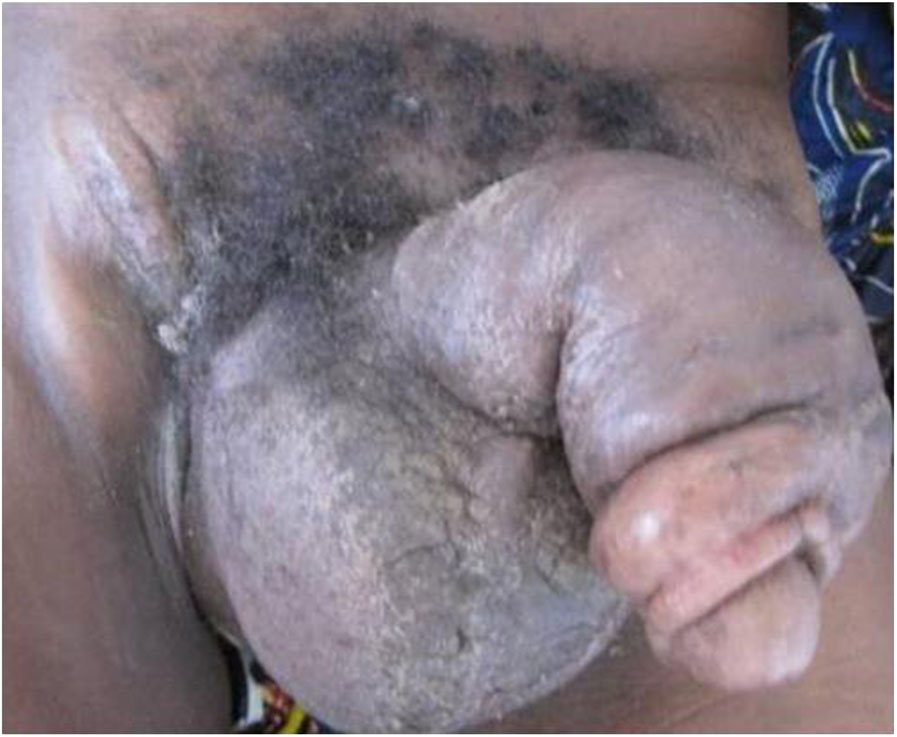
Dry whole perineal swelling with inguinal node involvement

A 22-year-old African man without specific medical history was admitted for a perineal mass evolving over 6 months, which was painful, and had appeared spontaneously. An examination found tenesmus, obstinate constipation, and dysuria. It revealed a dry perineal swelling, which was painless, and extended back to his anus, scrotum and penis. His testicles were mobile, and there were enlarged and fixed lymphadenopathies (Fig. 1).

A pelvic computed tomography (CT) scan found a large mass on his perineum, fairly limited, driving his testicles forward, with regional lymphadenopathies without secondary lesion.

**Fig. 2:**
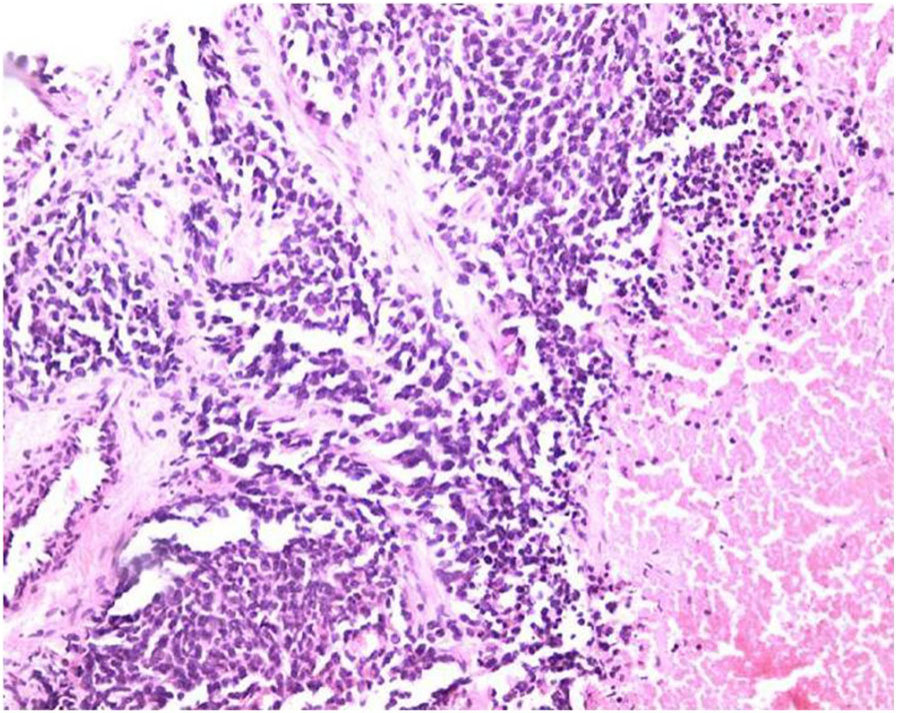
Increase in the nucleocytoplasmic ratio, monomorphic appearance, and eosinophilic cytoplasm appearance

A pathological examination of the biopsy fragments taken under ultrasound showed a tumor proliferation composed of small-sized cell nuclei with an increase in the nucleocytoplasmic ratio, with a fairly monomorphic appearance and associated with mitotic figures. These cells were disposed around slots. Some cells had an eosinophilic cytoplasm appearance (Fig. 2).

**Fig. 3:**
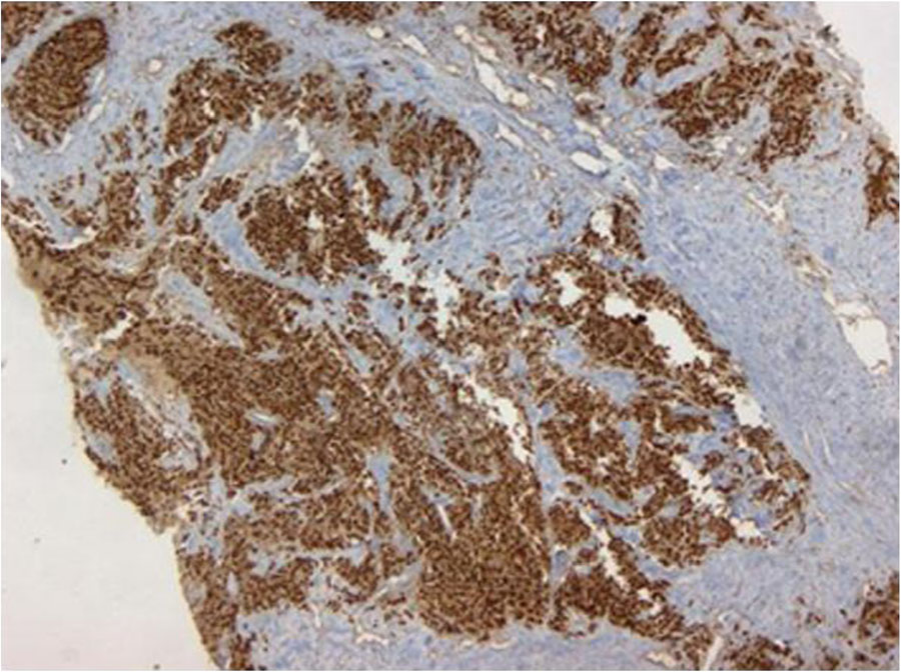
NCAM- and myogenin-positive coloration - low magnification

**Fig. 4:**
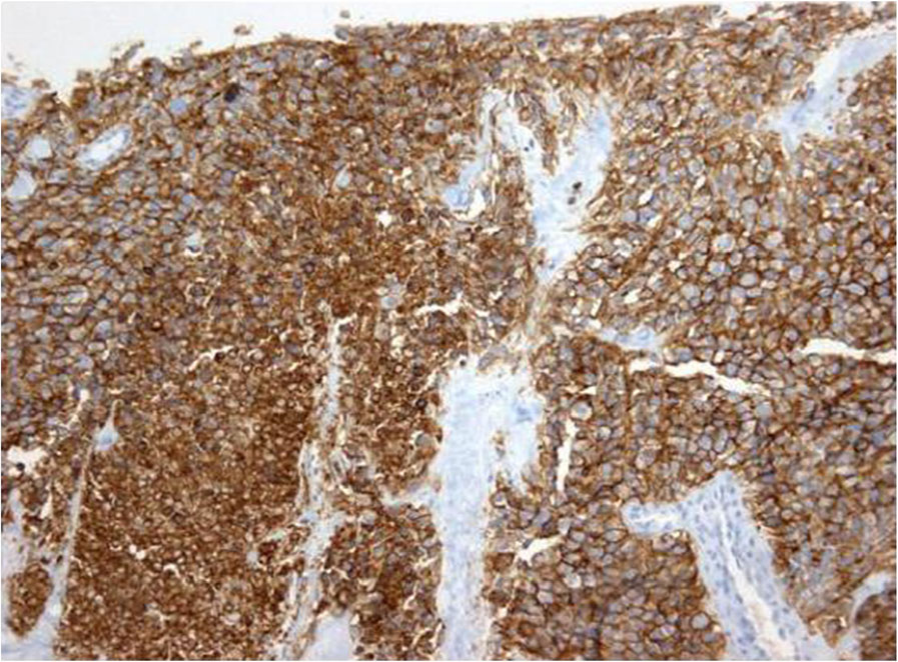
NCAM- and myogenin-positive coloration - high magnification

At immune marking, NCAM and myogenin (Figs. 3 and 4) were strongly positive, while CKAE1/AE3 antibody, CD45, synaptophysin, and chromogranin were negative. Our patient was classified T2N1M0 and then considered as stage III.

Outcome was quickly marked by anal obstruction and colostomy. He died after 6 months of follow-up with only supportive care.

## Discussion

Embryonal rhabdomyosarcoma is an extremely rare tumor with an estimated incidence of 0.45 to 0.69 cases per 100,000. This case is the first case in more than 20,000 patients in our practice. The incidence of RMS in general, and embryonic RMS especially, has a peak in young children between 0 and 4 years. The incidence decreases followed by another smaller peak between 15 and 19 years. After 19 years, the incidence decreases rapidly from 0.16 per 100,000 population between 20 and 24 years to 0.08 per 100,000 in the class of 25–29 years [1]. Definitive diagnosis is based on histological examination. A surgical biopsy is recommended. The needle biopsy is usually reserved for small lesions located in areas to be treated with radiation or chemotherapy or metastatic lesions [4]. The site of the lesion led us to a biopsy. CT and magnetic resonance imaging (MRI) morphological examinations are the most used in the expansion of the RMS balance sheet. A CT scan is advantageous for evaluation of bone lesions and abdominal lymphadenopathies. A MRI scan provides a better definition of the primary tumor and its relations with neighboring organs [4]. In our patient, CT was sufficient to characterize the lesion and examine the locoregional extension.

Embryonal RMS generally has an intermediate prognosis [2]. However, perineal locations have the worst predictions because they are usually diagnosed at a late stage [4]. The best prognostic factors in the perineal location are stage I with small size, less than 5 cm, absence of nodal involvement, and young age, less than 10 years [4]. The histological type in this location does not seem to impact overall survival [4].

The metastatic testicular damage in the ERMS has been rarely described. Gow *et al.* [5] reported two cases of metastatic testicular damage in ERMS in two young subjects [5]. In the case of our patient it is unclear whether the testicular damage was the result of contiguity or rather the metastatic disease.

For our patient, a temporary colostomy was indicated; it is often necessary in case of anal obstruction [4].

The oncological surgical treatment includes a complete resection of the original site with a circumferential margin of at least 0.5 cm [4]. In the anal and perineal locations, difficulties stem from the vicinity of the anal sphincter, urethra, and genitalia. In the case of our patient surgical resection was not considered because of the anal, penile, and scrotal involvement. Neoadjuvant chemotherapy seemed to be the best alternative.

Nowadays, almost all patients with ERMS receive chemotherapy [4]. The most widely used protocol combines vincristine, actinomycin D, and cyclophosphamide (VAC). New protocols based on ifosfamide and etoposide were used in combination with VAC in therapeutic trials with greater local control and better survival at 3 years than VAC only. These results suggest that the combination of ifosfamide-etoposide and VAC could be beneficial for advanced ERMS [6]. Other molecules such as irinotecan appear to be useful in the treatment of advanced ERMS [7]. After resection or biopsy, patients requiring radiotherapy are those having a microscopic or macroscopic residue. For many patients, radiation seems to be important to local disease control and survival [4]. At this localization, embryonal rhabdomyosarcoma seems to have better outcome than alveolar cases. Anal infiltration and lymph node involvement, as in our patient, are the main prognosis factors [8, 9].

## Conclusions

We reported a case of embryonal RMS extended to the perineum, scrotum, and penis with lymph node and testicular metastasis in 22-old-year African man. This is a rare condition. Treatment is based on surgery, chemotherapy, and radiotherapy, and prognosis depends on location and regional extension. Death occurs in the case of extended and nonresectable disease.

## Abbreviations

CD45: cluster of differentiation 45
CK AE1/AE3: cytokeratin AE1/AE3
CT: computed tomography
ERMS: embryonal rhabdomyosarcoma
MRI: magnetic resonance imaging
NCAM: neural cell adhesion molecule
VAC: vincristine, actinomycin D, and cyclophosphamide

## References

[1] Van Gaal JC, De Bont ES, Kaal SE, Versleijen-Jonkers Y, van der Graaf WT. Building the bridge entre rhabdomyosarcoma in children, teenagers and young adults: the road ahead. Crit Rev Oncol Hematol. 2012;82(3):259–79. 10.1016/j.critrevonc.2011.06.005 21802959

[2] Newton Jr WA, Gehan EA, Webber BL, Marsden HB, van Unnik AJ, Hamoudi AB, *et al.* Classification of rhabdomyosarcomas and related sarcomas. Pathologic aspects and proposal for a new classification-Intergroup Rhabdomyosarcoma Study year. Cancer. 1995;76(6):1073–85. 10.1002/1097-0142(19950915)76:6<1073::aid-cncr2820760624>3.0.co;2-l 8625211

[3] Pappo AS, Shapiro DN, Crist WM, Maurer HM. Biology and therapy of pediatric rhabdomyosarcoma. J Clin Oncol. 1995;13(8):2123–39. 10.1200/JCO.1995.13.8.2123 7636557

[4] Leaphart CD, Rodeberg D. Pediatric surgical oncology: management of rhabdomyosarcoma. Surg Oncol. 2007;16(3):173–85. 10.1016/j.suronc.2007.07.003 17689957

[5] Gow KW, Murphy JJ, Wu JK, Desa DJ. Metastatic testicular rhabdomyosarcoma - a report of two cases. J Pediatr Surg. 2003;38(8):E1–3. 10.1016/s0022-3468(03)00288-4 12891513

[6] Sandler E, Lyden E, Ruymann F, Maurer H, Wharam M, Parham D, Link M, Crist W. Efficacy of ifosfamide and doxorubicin given as a phase II “window” in children with newly diagnosed metastatic rhabdomyosarcoma: a report Intergroup Rhabdomyosarcoma Study Group. Med Pediatr Oncol. 2001;37(5):442–8. 10.1002/mpo.1227 11745872

[7] Pappo AS, Lyden E, Breitfeld P, Donaldson SS, Wiener E, Parham D, *et al.* Two consecutive phase II trials of irinotecan window alone or in combination with vincristine for the treatment of metastatic rhabdomyosarcoma: the Children’s Oncology Group. J Clin Oncol. 2007;25(4):362–9. 10.1200/JCO.2006.07.1720 17264331

[8] Casey DL, Wecler LH, LaQualigli MP, Meyers PA, Wolden SL. Patterns of failure for rhabdomyosarcoma of the perineum and perianal region. Int J Radiat Oncol Biol Phys. 2014;89(1):82–7. 10.1016/j.ijrobp.2014.01.051 24725692

[9] Fuchs J, Dantonello TM, Blummenstock G, Koztyla D. Treatment and outcome of patients suffering from perineal/perianal rhabdmyosarcoma: results from the CWS trials-retrospective clinical study. Ann Surg. 2014;259(6):1166–72. 10.1097/SLA.0b013e3182a6f320 24045440

